# Retigeric Acid B Attenuates the Virulence of *Candida albicans* via Inhibiting Adenylyl Cyclase Activity Targeted by Enhanced Farnesol Production

**DOI:** 10.1371/journal.pone.0041624

**Published:** 2012-07-23

**Authors:** Wenqiang Chang, Ying Li, Li Zhang, Aixia Cheng, Hongxiang Lou

**Affiliations:** School of Pharmaceutical Sciences, Shandong University, Jinan City, Shandong Province, China; Université de Nice-CNRS, France

## Abstract

*Candida albicans*, the most prevalent fungal pathogen, undergoes yeast-to-hyphal switch which has long been identified as a key fungal virulence factor. We showed here that the lichen-derived small molecule retigeric acid B (RAB) acted as an inhibitor that significantly inhibited the filamentation of *C. albicans,* leading to the prolonged survival of nematodes infected by *C. albicans*. Quantitative real-time PCR analysis and intracellular cAMP measurement revealed RAB regulated the Ras1-cAMP-Efg1 pathway by reducing cAMP level to inhibit the hyphae formation. Confocal microscopic observation showed RAB induced the expression of Dpp3, synthesizing more farnesol, which was confirmed by gas chromatography-mass spectroscopy detection. An adenylyl cyclase activity assay demonstrated RAB could repress the activity of Cdc35 through stimulating farnesol synthesis, thus causing a decrease in cAMP synthesis, leading to retarded yeast-to-hyphal transition. Moreover, reduced levels of intracellular cAMP resulted in the inhibition of downstream adhesins. Together, these findings indicate that RAB stimulates farnesol production that directly inhibits the Cdc35 activity, reducing the synthesis of cAMP and thereby causing the disruption of the morphologic transition and attenuating the virulence of *C. albicans*. Our work illustrates the underlying mechanism of RAB-dependent inhibition of the yeast-to-hyphal switch and provides a potential application in treating the infection of *C. albicans*.

## Introduction

*Candida albicans*, an opportunistic human pathogen, causes systemic candidiasis mainly in immunocompromised individuals, with an estimated 40% mortality rate [1]. The success of *C. albicans* as the leading fungal pathogen is a result of its virulence factors such as adhesins, phenotypic switch, secreted aspartyl proteases or phospholipases, among which the switch from yeast-to-hyphal was more concerned [2]. The yeast-to-hyphal transition, and upregulation of hyphal-specific genes during the course of infection increase the virulence potential [3]. Filaments have the ability to promote tissue penetration and escape from immune cells, which results in systemic infection accompanied with yeast-form dissemination [4].

For *C. albicans*, morphogenesis was regulated by a small GTPase, Ras1, that controls two intricate pathways including cyclic AMP (cAMP)-dependent protein kinase A (PKA) pathway and mitogen-activated protein kinase (MAPK) Cascade [5]. The genes and related proteins that regulate the morphologic switch are important targets for the discovery of antifungal therapeutics. Farnesol, a quorum sensing molecule, secreted by *C. albicans* could regulate the yeast-to-hyphal transition and biofilm formation [6], [7]. Dpp3 acts as a phosphatase to convert farnesyl pyrophosphate to farnesol [8]. Therefore agents which could induce the Dpp3 expression, stimulating more farnesol synthesis, have potential antifungal effect.

Lichens, the symbiotic organisms consisting of fungi and algae, produce abundant secondary metabolites with multiple bioactivities such as antibiotic, antifungal, antiviral, and antiinflammatory properties [9]. We have previously shown that retigeric acid B (RAB), a pentacyclic triterpene acid from the lichen species *Lobaria kurokawae*, had antifungal activity when applied alone or in combination with azoles, especially to azoles-resistant strains [10].

In the present study, we showed that RAB inhibited the yeast-to-hyphal transition of *C. albicans in vitro*. *In vivo* RAB conferred the prolonged survival of *C. elegans* infected with *C. albicans* through inhibiting the hyphae formation. We also uncovered that RAB stimulated farnesol production, which repressed the activity of Cdc35, causing the defect of intracellular cAMP synthesis, thereby inhibiting the morphologic switch of *C. albicans*.

**Table 1:** Gene-specific primers used for qPCR.

Primers	sequence (5′→3′)
*GSP1-F*	TGAAGTCCATCCATTAGGAT
*GSP1-R*	ATCTCTATGCCAGTTTGGAA
*RAS1-F*	GGCCATGAGAGAACAATATA
*RAS1-R*	GTCTTTCCATTTCTAAATCAC
*CDC35-F*	TTCATCAGGGGTTATTTCAC
*CDC35-R*	CTCTATCAACCCGCCATTTC
*PDE2-F*	ACCACCACCACTACTACTAC
*PDE2-R*	AAAATGAGTTGTTCCTGTCC
*EFG1-F*	TATGCCCCAGCAAACAACTG
*EFG1-R*	TTGTTGTCCTGCTGTCTGTC
*TEC1-F*	AGGTTCCCTGGTTTAAGTG
*TEC1-R*	ACTGGTATGTGTGGGTGAT
*CST20-F*	TTCTGACTTCAAAGACATCAT
*CST20-R*	AATGTCTATTTCTGGTGGTG
*HST7-F*	ACTCCAACATCCAATATAACA
*HST7-R*	TTGATTGACGTTCAATGAAGA
*CEK1-F*	AGCTATACAACGACCAATTAA
*CEK1-R*	CATTAGCTGA ATGCATAGCT
*CPH1-F*	ATGCAACACTATTTATACCTC
*CPH1-R*	CGGATATTGTTGATGATGATA
*ALS3-F*	CTAATGCTGCTACGTATAATT
*ALS3-R*	CCTGAAATTGACATGTAGCA
*HWP1-F*	TGGTGCTATTACTATTCCGG
*HWP1-R*	CAATAATAGCAGCACCGAAG
*ECE1-F*	GCTGGTATCATTGCTGATAT
*ECE1-R*	TTCGATGGATTGTTGAACAC

## Methods

### Strains and Growth Conditions

*C. albicans* isolates YEM30, SC5314, CA2, CA10 and CASA1 [11] were used in this study and stored in culture medium supplemented with 10% (v/v) glycerol at −80°C. The isolates were propagated in YPD medium agar plates at 30°C and then inoculated into liquid YPD medium and incubated overnight in an orbital shaker with 100 rpm at 30°C. In each assay, RPMI1640 medium was added with 1% glucose except for MIC determination. *Caenorhabditis elegans glp-4*; *sek-1* strain was purchased from the Caenorhabditis Genetics Center, which is funded by the NIH National Center for Research Resources. The *C. elegans* was propagated on *E. coli* strain OP50 and cultured using previously described methods [12]. Human epidermoid carcinoma VCR-selected KB/VCR cell line [13], [14] with characteristics of MDR attributable to expression of P-gp (a gift from Dr. J. Ding, Shanghai Institute of Materia Medica, Chinese Academy of Sciences, Shanghai, China) was used in this study and cultured as previously described [15]. The KB cell line was originally obtained from the American Type Culture Collection (Rockville, MD).

**Table 2:** The antifungal efficacy of RAB *in vitro* and *in vivo* with FLC as positive control.

Strain	FLC	RAB	FLC	RAB
	MIC_80_ *in vitro* (µg/ml )		EC_50_ *in vivo* (µg/ml )	
YEM30^a^	2	8	2	4
SC5314	2	8	4	8
CASA1^b^	1	8	2	2
CA2^c^	0.5	16	2	16
CA10^c^	256	16	8	16

a MIC_80_ *in vitro* has been reported [41], [42].

b MIC_80_ of FLC *in vitro* has been reported [43].

c MIC_80_ *in vitro* has been reported in our lab [10].

### Antifungal Agents

RAB was isolated from the lichen *L. kurokawae* in our laboratory with its purity over 97% analyzed by high-performance liquid chromatography; Fluconazole (FLC) was obtained from the Institute of Biopharmaceuticals of Shandong. China. The agents were prepared using DMSO with its content below 0.5% in each assay.

### Antifungal Assay *In vitro* and *In vivo*

The MIC_80_ was determined by the broth microdilution procedure recommended by the Clinical and Laboratory Standards Institute (formerly the National Committee for Clinical Laboratory Standards) [16] and the *C. elegans–C. albicans assay* was conducted to determine the EC_50_, conferring 50% survival of the worms using previously described method [12], [17]. In *C. elegans*–*C. albicans* assay, nematodes were grown on nematode growth medium (NGM) with *Escherichia coli* strain OP50 as the food source. And then they were washed with M9 buffer and placed on 48 h-old *C. albicans* lawns (on BHI agar plates) for 2 h. The worms were washed off the plates with screen medium and re-suspended at a density of 1–2 worm/µl in screen medium. 20 µl of the suspension of pre-infected worms were added to wells of 96-well plates (Corning). 80 µl of screen medium containing drugs was dispensed into the indicated well. The survival rates were calculated by counting the live and dead worms based on nematode shape, as live worms appear sinusoidal and dead worms are rod shaped. To image the differences between drug-treated nematodes and non-treated ones, the experiments were conducted as follows. After 5 days *C. elegans*–*C. albicans* (CASA1) coinoculation, the plates were imaged using an Olympus microscope equipped with a 4× magnification objective lens. The resulting images were visually analyzed for *in vitro* fungal growth. Then the nematodes were transferred to a glass slide and observed with a Zeiss LSM 700 Meta laser scanning confocal microscope. Bright field and fluorescent images were taken. The argon laser (488 nm) and band-pass filter (500–560 nm) were set up for GFP observation.

**Figure 1:**
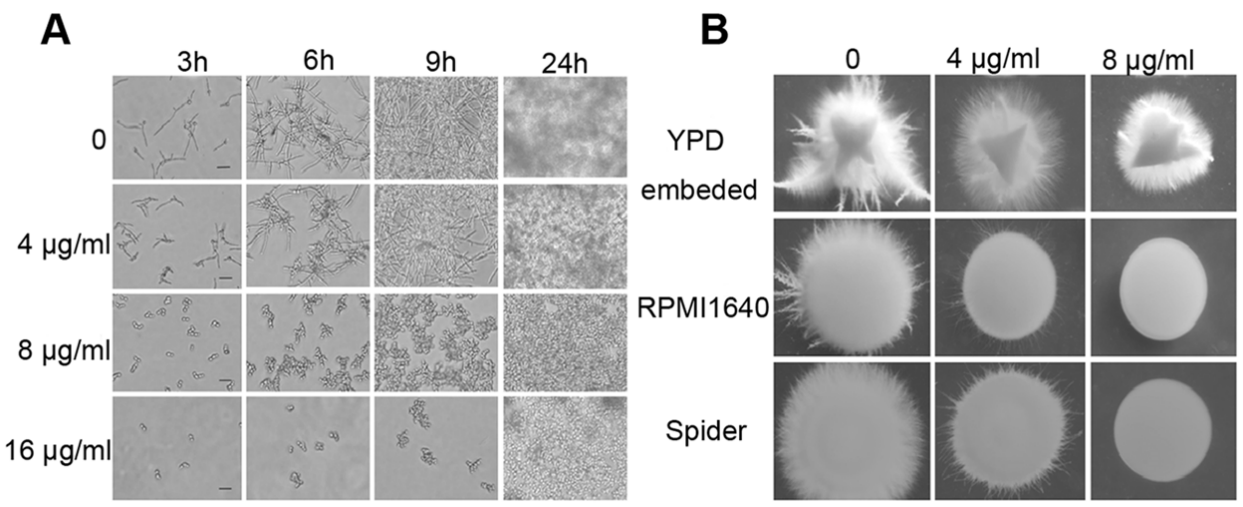
The inhibitory effect of RAB on hyphae formation of *C. albicans* YEM30 *in vitro*. (A) Yeasts were cultured in liquid RPMI1640 medium containing a series of concentrations of RAB. At the indicated time the images were captured by a light field microscope (Scale bars: 20 µm). (B) The hyphae formation was inhibited by RAB at the indicated solid media. The images were photographed with 40× magnification after 5 days of incubation at 30°C.

**Figure 2:**
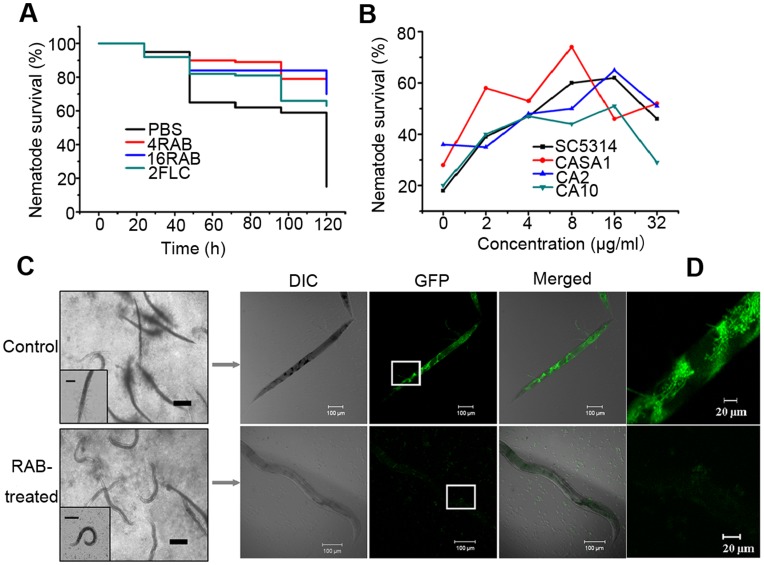
RAB confers improved nematodes’ survival by retarding the formation of invasive hyphae. (A) Nematodes were infected with *C. albicans* YEM30 for 2 h and then moved to pathogen-free liquid media in the presence of PBS (negative control), RAB (4 or 16 µg/ml) or 2 µg/ml of FLC (positive control). Each day the worms were monitored and the survival rate was calculated. *p*<0.001 for 4RAB, 16RAB and FLC treated groups compared to PBS-treated group. (B) Nematodes were infected with the indicated *C. albicans* strains and then treated by a series of doses of RAB. After 5 days of therapy, the survival ratio in each group was calculated. (C) The nematodes were infected by *C. albicans* strain CASA1 with *GFP* tagged *CDR1* for 2 h and then moved into pathogen-free liquid media containing drugs or the vehicle for 5 days. And then they were observed in 4× objective magnification. Most nematodes in control group did not demonstrate any movement and developed filaments outside the body. However, the majority of the drug-treated were alive and not invaded by the hyphae (scale bars: 200 µm). Insert shows the images of nematodes captured at 200× magnification (scale bars: 100 µm). The representative worm was selected for confocal microscopic observation. Green fluorescence of hyphae cells and yeast cells was seen within the intestine of the dead nematode under the vehicle treatment. And no *C. albicans* cells were observed in the live worm challenged by RAB (about 60 nematodes in each group). (D) Enlarged view of the rectangle frame.

### Morphologic Transition Test of *C. albicans*

The strain YEM30 (2×10^5^ cells/ml in RPMI1640 medium) was incubated with different concentrations of RAB in 96-well flat-bottomed microtitration plates at 37°C without shaking. At the indicated time, the cells were photographed with an Olympus fluorescent microscope (Olympus IX71, Olympus, Tokyo, Japan). YEM30 cells were also cultured in liquid RPMI1640 medium plus 25 mM sodium bicarbonate, 5% CO_2_ or in a closed-system (creating a hypoxia condition) with various concentrations of RAB. After 3 h, the morphology was observed using a light-field microscopy. The yeast-to-hyphal conversion regulated by RAB was also performed on three different solid media as described below. The YEM30 cells were diluted to 10^3^ cells/ml and then aliquot 50 µl was evenly smeared on the RPMI1640 or Spider agar plates (with its liquid media mixed with 1.5% agar) containing different concentrations of RAB. For the embedded condition test, the diluted cells were mixed with melting YPD agar containing RAB. All the agar plates were incubated at 30°C and the resulting colonies were photographed by microscopy with 40× magnification after 5 days. These experiments were done in triplicate on a given day and repeated on separated days.

**Figure 3:**
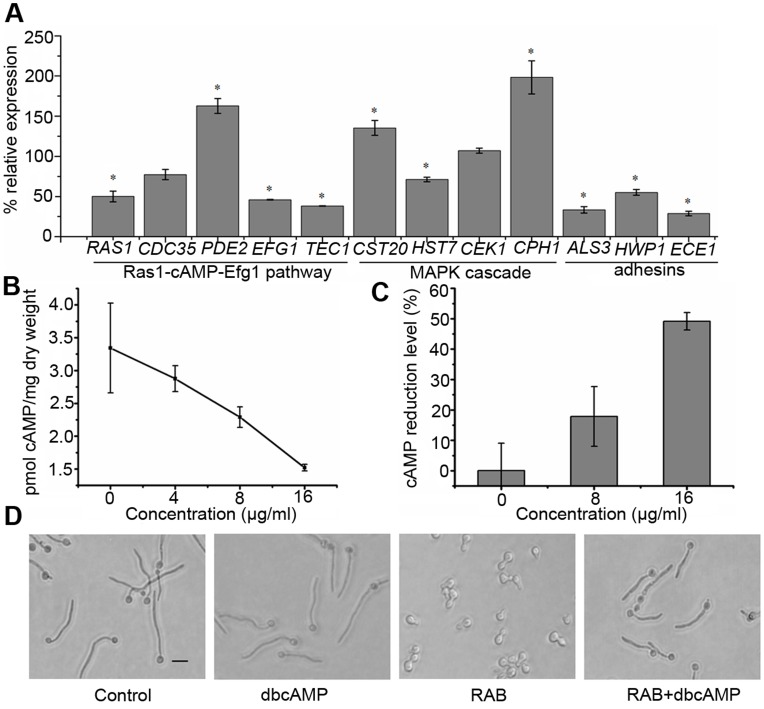
Reduction of cAMP confers the defect of hyphae formation induced by RAB. (A) The expressions of hyphae-specific genes in response to RAB treatment. *C. albicans* YEM30 was cultured in RPMI1640 medium with or without 10 µg/ml RAB for 8 h at 37°C and then cells were harvested for RNA extraction. Genes involving in modulating hyphae formation or hyphae-specific were detected for transcript levels using qPCR. The expression of detected genes was normalized to *GSP1* and relative to the control group using formula 2^−ΔΔCT^. The values are means ± standard deviations of three independent experiments. Asterisk (*) represents significance with *p*<0.05. (B–C) Reduced intracellular cAMP level by RAB. YEM30 cells were cultured in RPMI1640 medium containing a series of RAB or the vehicle for 8 h (B) or 16 h (C) at 37°C. Cells were harvested for cAMP assay. (D) Exogenous cAMP restores the RAB-inhibited hyphae formation. YEM 30 cells were grown in RPMI1640 medium with the indicated treatment. After 3 h, the morphology was visualized by microscopy (the scale bar: 20 µm).

### qPCR Analysis

The expressions of hyphae formation related genes were measured using real-time PCR after treatment with RAB. *C. albicans* YEM30 cells (2×10^5^ cells/ml in RPMI 1640) were incubated with or without RAB (10 µg/ml) for 8 hours. The total RNAs were respectively isolated using the hot phenol method as previously described [18] and converted to cDNA using the RT, ReverTraAce (Toyobo Co., Osaka, Japan). PCR reactions were conducted with SYBR Green (Toyobo Co., Ltd.) in an Eppendorf Mastercycler Real Time PCR System with preliminary denaturation for 2 min at 95°C, followed by 40 amplification cycles of denaturation at 95°C for 20 s, annealing at 60°C for 20 s, and primer extension at 72°C for 10 s. Primers used in this assay are shown in Table 1. GSP1, which was not transcriptionally regulated in the morphogenesis switch, served as the internal control. And the transcript level of detected genes was calculated using the formula 2^−ΔΔCT^.

**Figure 4:**
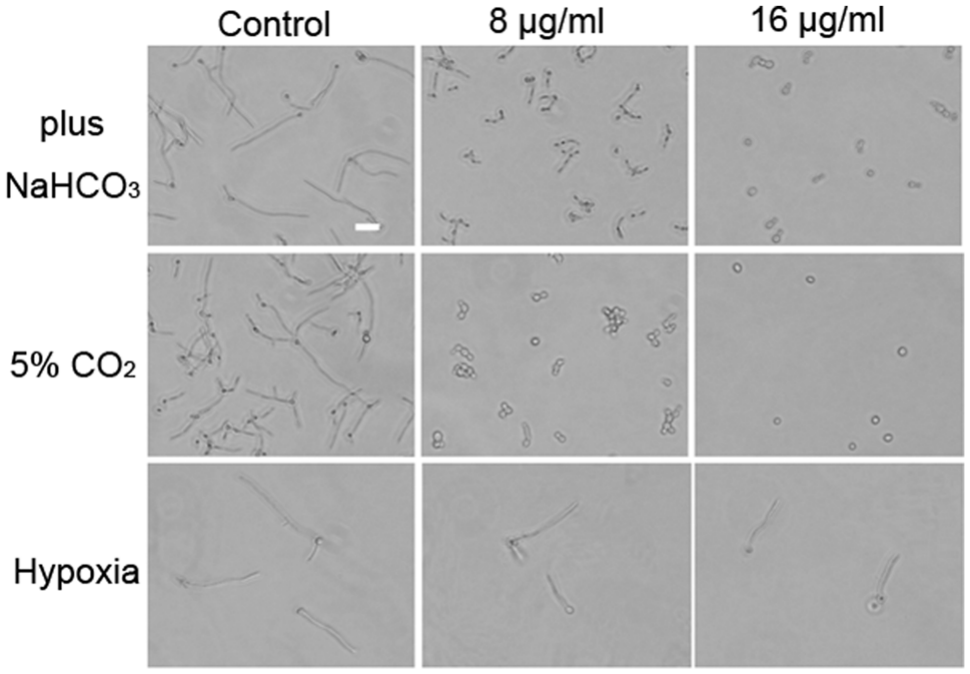
The morphology of *C. albicans* YEM30 challenged by RAB at the indicated conditions. The bar: 20 µm.

**Figure 5:**
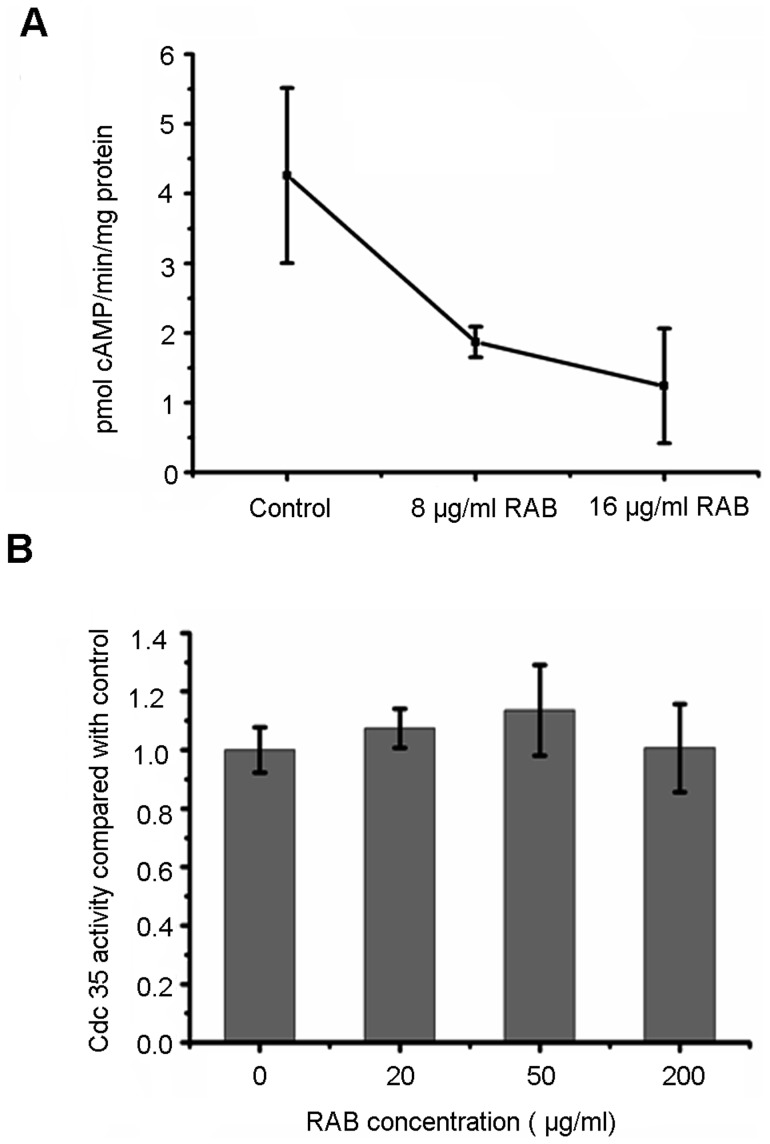
The activity of Cdc35 is indirectly inhibited by RAB. (A) YEM30 cells were subject to the indicated RAB treatment for 8 h and then were broken by beads beating. The lysates were assayed for the activity of Cdc35 by detecting the cAMP production as described in METHODS. (B) The purified catalytic domain of *C. albicans* Cdc35 was incubated with the indicated concentration of RAB for 20 min, then cAMP production was measured to determine the activity of Cdc35 under the treatment of RAB.

### Measurement of Intracellular cAMP Level

*C. albicans* YEM30 (2×10^5^ cells/ml) were cultured in RPMI1640 medium for 8 h or 16 h in the presence of antifungal agents. And intracellular cAMP was extracted as previously described [19] and quantified using a Monoclonal Anti-cAMP Antibody Based Direct cAMP ELISA Kit (NewEastBiosciences).

### cAMP Rescue Experiments

Grown overnight *C. albicans* cells were diluted to 2×10^5^ cells/ml with RPMI1640 medium. Dibutyryl-cAMP (Sigma) was added to the cultures with a final concentration of 5 mM immediately following the addition of 8 µg/ml RAB. The non-RAB treatment cells with or without dibutyryl-cAMP (dbcAMP) served as control. At the indicated time, cells were visualized by an Olympus microscope.

**Figure 6:**
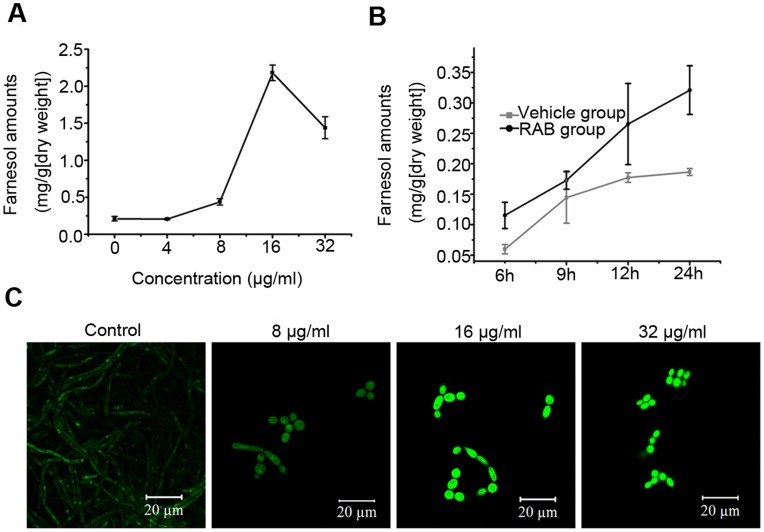
Farnesol and Dpp3 are stimulated by RAB in a dose-dependent manner. (A) *C. albicans* YEM30 was cultured in RPMI1640 medium containing RAB for 12 h, and farnesol in the supernatants was extracted and quantified by GC-MS. (B) The comparison of farnesol secretion of YEM30 strain at the indicated time between the vehicle group and RAB-treated one (10 µg/ml). (C) The expression of Dpp3 induced by RAB was observed using *DPP3-GFP-*BWP17 strain by confocal microscopy.

### Adenylyl Cyclase Activity Assay

The adenylyl cyclase activity was measured in two independent ways. One method was performed as previously described with some modification [20]. Briefly, fragments encoding amino acids 1166–1571 of Cdc35 (containing catalytic domain of Cdc35) were cloned into pET-32a(+) and the His-Cdc35 fusion protein was expressed in *E. coli* BL21 and purified using Mag Extractor-His- tag kit (Toyobo, Tokyo, Japan). Cyclase assays were performed in a final volume of 100 µl, with ∼200 ng of purified His-Cdc35 in the presence of 50 mM Tris-HCl (pH 7.5), 10 mM ATP, and 10 mM MgCl_2_, 10 mM creatine phosphate, 50 U/ml creatine phosphokinase and different concentrations of RAB. Reactions were incubated at 37°C for 20 min and stopped by addition of 100 µl of 0.2 N HCl. Then cAMP was detected using a Monoclonal Anti-cAMP Antibody Based Direct cAMP ELISA Kit. The other method was conducted as described before with slight alteration [21]. Briefly YEM30 was cultured in RPMI1640 medium under the treatment of 8 or 16 µg/ml of RAB at 37°C for 8 h. Subsequently cells were harvested by centrifugation and resuspended in ice-cold 25 mM Tri-HCl (pH 7.5). After two washes, the cells were resuspended in 500 µl of the same buffer in a 2 ml screw-cap tube and mixed with 500 µl of acid-washed glass beads. Cells were lysed on an automated Precellys 24 bead-based homogenizer (Bertin Technologies, Montigny-le-Bretonneux, France) at 5500 rpm for 9 rounds of 1.5 min beating with cooling on ice between rounds. Then 100 µl of the lysate was mixed with 100 µl of reaction mix containing 25 mM Tris-HCl (pH 7.5), 5 mM MgCl_2_, 20 mM creatine phosphate, 100 U/ml creatine phosphokinase, and 10 mM ATP or not. The reaction was incubated at 37°C for 15 min and then terminated by adding 200 µl of 0.2 N HCl. Protein was detected using a BCA kit and cAMP contents in each group were detected using a cAMP ELISA Kit. The cAMP amount produced by the lysates was calculated as follows: the cAMP level in the reactive solution containing substrate ATP subtracting the one without ATP is divided by the protein amount. All experiments were performed in triplicate for each condition and repeated three times.

**Figure 7:**
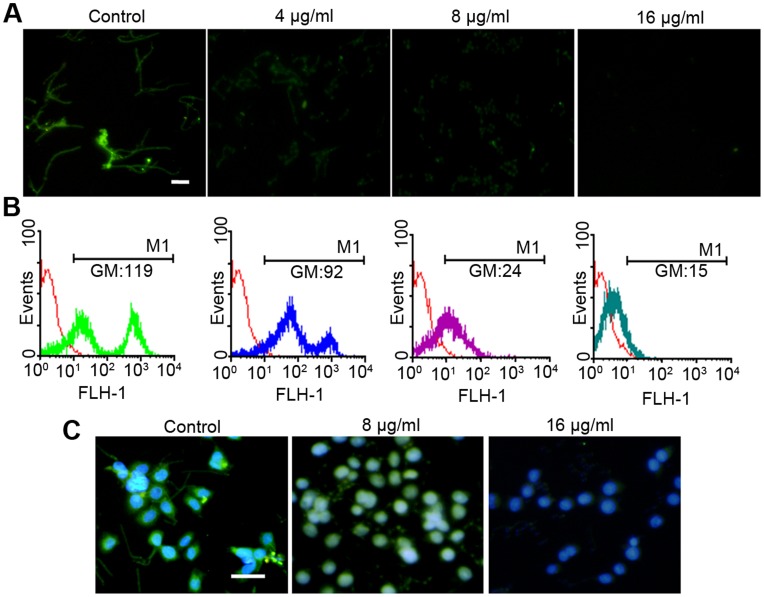
The expression of Als3, an invasin, is reduced by RAB. (A–B) *C. albicans ALS3-GFP*-BWP17 cells were cultured in RPMI1640 medium containing the indicated concentrations of RAB. After 3 h incubation, the cells were stained using indirect immunofluorescence as described in Methods. And the fluorescence was measured using fluorescence microscopy (A) as well as flow cytometry (B) (The scale bar: 20 µm). The expression of Als3 was indicated by the geometric mean (GM) of fluorescence intensity. BWP17 strain served as negative control which was indicated by red line. (C) The KBV cell line was coinoculated with *C. albicans ALS3-GFP*-BWP17 cells for 3 h. Subsequently cells were processed following immunofluorescence method to enlarge the fluorescence intensity of GFP, and then stained with DAPI. Finally cells were observed using a fluorescence microscope (The bar: 50 µm).

### Quantification of Farnesol Secreted in the Supernatant

*C. albicans* was cultured in RPMI1640 medium and treated by RAB. The solvent of RAB served as control. Cells in each group were collected at the indicated time and the supernatants were transferred to new tubes and added with a certain amount of internal standard, 4-hydroxybenzyl alcohol. Farnesol in the supernatants of culture was extracted with the same volume of ethyl acetate and quantified using GC-MS as previously described with some modification [22]. Briefly the supernatants were sterilized with 0.22-µm (pore-size) cellulose nitrate filters and extracted with ethyl acetate. The active fractions were resuspended in 100% acetone and analyzed by a Waters GCT Premier gas chromatography-mass spectroscopy (GC-MS) (Waters Corporation, Milford, MA) with a 30-m DB-35 column in electron ionization (EI) mode. GC used a 1-µl sample, injector, and detector temperatures of 230 and 280°C, respectively, and a temperature program of 100°C for 3 min and then 20°C/min until 280°C for 2 min. MS used a 3-min solvent delay and a scan rate of 1.5 scans/s.

### Measurement of Dpp3 Expression Under the Treatment of RAB

BWP17*-DPP3-GFP* [23] was cultured in RPMI1640 medium in 24-well flat-bottomed microtitration plates at 37°C and treated with different concentrations of RAB for 12 h. The cells were scraped from the wells and observed using a Zeiss LSM 700 Meta laser scanning confocal microscope.

### Construction of Green Fluorescent Protein-tagged *C. albicans* Strain

The *C. albicans* strain *ALS3-GFP*-BWP17 (MG1003) was created by homologous recombination of green fluorescent protein (GFP) sequences into the 3′ end of their open reading frame using the methods as previously described [24]. The DNA used for the transformation was created by PCR using primers containing ∼70 base pairs of sequence homologous to the 3′ end of the *ALS3* open reading frame to amplify a cassette containing GFP and a HIS1 selectable marker using pGFP-HIS1 as the template. The primer sequences used were 5′-CGGATCTGGTTCTGTTATTCAACATTCTACTTGGTTATGTGGTTTGATCACATTATTATCCTTATTTATTGGTGGTGGTTCTAAAGGTGAAGAATTATT-3′ and 5′-CCTGAAACTGTACAAGCGATGCATAACCTCAAGTAAAGATTATATTACAATAAATTCCAGAGTCGAATTCCGGAATATTTATGAGAAAC-3′. The PCR product was transformed into auxotrophic mutant strain BWP17. The transformed strain was then spotted on SD minus histidine (SD−His) solid medium and grown at 37°C for 3 d. The colonies resulting from the transformation were then screened for GFP-positive cells by fluorescent microscopy.

### Assays of Detecting the Expression of Als3

*ALS3-GFP*-BWP17 was diluted to 2×10^5^ cells/ml with RPMI1640 medium in 24-well flat-bottomed microtitration plates at 37°C. RAB was added with two-fold increased concentrations. After a 3 hour culture, the cells were collected and washed twice with PBS, then fixed with 4% paraformaldehyde for 15 min, and incubated for 1 h in PBS with 1% bovine serum albumin (BSA). Subsequently, the fixed cells were washed three times with PBS and incubated with mouse anti-GFP monoclonal antibody (Cell Signaling Technology, Beverly, MA) diluted 1∶200 in PBS with 1% BSA for 1 h at room temperature. After washing, the cells were incubated for another 1 h with a goat anti-mouse secondary antibody conjugated with Alexa 488 (Invitrogen) diluted 1∶100. Finally, the cells were washed three times with PBS, detached from the coverslips using a pipet point and resuspended in 0.5 ml PBS. The cells were observed with an Olympus fluorescent microscope and also detected by flow cytometry. To measure the expression of Als3 when *C. albicans* invaded the epithelial cells, KBV cells were grown on 12 mm glass coverslips for 24 h post seeding and then infected with *C. albicans* strain *ALS3-GFP*-BWP17. After 3 h, the next procedures were followed as described above except that cells were stained with DAPI before detached from coverslips. Finally cells were observed by fluorescent microscopy.

### Statistical Analysis

The experimental data were statistically analyzed using Student’s *t*-test. *p*<0.05 was considered significant.

## Results

### The Antifungal Efficacy of RAB *In vitro* and *In vivo*

We first tested the antifungal activity of RAB against *C. albicans* strains SC5314, YEM30, CASA1, CA2 and CA10 according to the CLSI standard [16]. FLC was used as control (Table 2). We found that RAB displayed antifungal activity with 8 or 16 µg/ml of MIC_80_ against these strains of *C. albicans in vitro*. We then evaluated the efficacy and toxicity of RAB using the nematode *C. elegans* followed the methods described elsewhere [12] *in vivo.* The EC_50_ of RAB against these *C. albicans* isolates ranged from 2 µg/ml to 16 µg/ml (Table 2).

### RAB Attenuates Virulence through Inhibiting Hyphae Formation

Hyphae formation, as a virulent determinant, plays a vital role in the pathogenesis. We next examined the effect of RAB on the yeast-to-hyphal switch *in vitro*. RAB inhibited the filamentation of *C. albicans* at 8 µg/ml within 24 hours (Fig. 1A), while untreated *C. albicans* cells formed germ tubes at the beginning followed by extensive hyphal networks by 24 h. In addition, RAB also inhibited the filamentation in a dose-dependent fashion when *C. albicans* was grown on either Spider or RPMI1640 agar medium, although the inhibitory effects in embedded condition were less significant (Fig. 1B). *In vivo* experiments with *C. elegans* also found that RAB provided considerable protection to the worms through preventing hyphae formation (Fig. 2). RAB (4 or 16 µg/ml) conferred up to 70% survival of the nematodes after 5 days of infection with the YEM30 strain (Fig. 2A). And RAB (2 to 32 µg/ml) also prolonged the worms’ survival when infected with other *C. albicans* strains (Fig. 2B). However, high concentration of RAB (32 µg/ml) displayed some toxicity for nematodes (Fig. 2B). We then utilized a GFP-labeled strain CASA1 to observe the filamentation in the cuticle or within the nematode. The images showed the filaments penetrated the nematode cuticle and then initiated biofilm formation within the intestine in untreated worms. However, infected nematodes treated with RAB showed no hyphae (Fig. 2 C and D). This suggests RAB could reduce the pathogenesis of *C. albicans* through inhibiting the formation of invading hyphae.

### RAB Regulates the Ras1-cAMP-Efg1 Pathway by Reducing Intracellular cAMP to Inhibit the Hypahe Formation

To uncover the underlying mechanism by which RAB inhibited hyphae formation, we investigated the expression of genes in the Ras1-cAMP-Efg1 and MAPK signal pathways with quantitative real-time PCR (qPCR). We found that genes in the Ras1-cAMP-Efg1 pathway in the early growth stage were downregulated by RAB, while *PDE2*, encoding phosphodiesterase, was upregulated (Fig. 3 A). *CST20* and *CPH1* in the MAPK cascade were upregulated as a kind of feedback for the inhibited Ras1-cAMP-Efg1 pathway (Fig. 3 A). As cAMP is a critical element regulating morphogenesis, we next determined the intracellular cAMP contents in cells treated with RAB for 8 h or 16 h. The data showed that RAB reduced intracellular cAMP level (Fig. 3 B and C), and thereby caused the retarded yeast-to-hyphal transition, which was confirmed by exogenous cAMP rescue experiments (Fig. 3 D). The results demonstrate RAB actually reduces the intracellular cAMP level, which regulates the Ras1-cAMP-Efg1 to inhibit yeast-to-hyphal transition.

### The Activity of Cdc35 is Indirectly Inhibited by RAB

To further indentify the key element RAB affected, we performed investigations based on decreased cAMP level. Previous research reported CO2/bicarbonate could activate Cdc35 and bypass Ras in *C. albicans* [20], [25]. Thus, an experiment was designed to observe the anti-hyphae effect of RAB in the absence or presence of 5% CO2 and/or 25 mM sodium bicarbonate. No differences were observed between absence and presence of CO2/bicarbonate (Fig. 4). The data suggested that RAB had no direct interaction with Ras1. Interestingly, we also found that RAB lost its inhibitory function against hyphae formation when incubating with *C. albicans* cells under hypoxic condition, which was consistent with the observation in embedded condition (Fig. 1B and Fig. 4). Because Efg1 was a major regulator in response to hypoxic condition [26], it was inferred that RAB had no effect on Efg1. Based on the aforementioned analysis of Ras1-cAMP-Efg1 pathway, we predicted RAB might affect the activity of Cdc35, the enzyme synthesizing cAMP, although our previous report showed RAB depleted the ATP, the substrate of cAMP synthesis. Then we performed the adenylyl cyclase activity assay to detect the activity of Cdc35 under the treatment of RAB. We treated *C. albicans* cells with RAB and then lysed the treated cells to determine the Cdc35 activity in the cell lysates. Our results showed the activity of Cdc35 was repressed when treated by RAB at the dose of 8 or 16 µg/ml after 8 h (Fig. 5 A). To determine whether RAB directly inhibit Cdc35, the purified catalytic domain of Cdc35 was used in the adenylyl cyclase activity inhibition assays. Experimental results suggested RAB did not influence the enzymatic activity (Fig. 5 B).The above data suggest another factor induced by RAB may inhibit the activity of Cdc35.

### Farnesol Secretion of *C. albicans* Stimulated by RAB

Hornby et al. proposed a general theme that inhibition of the sterol pathway would lead to increased production of farnesol [27], [28], a quorum sensing molecule that could inhibit the yeast-to-hyhae conversion of *C. albicans* [7], [29]. A recent study showed farnesol could directly inhibit the activity of Cdc35 [30]. Based on our previous result of decreased ergosterol content induced by RAB [31], we thus utilized gas chromatography−mass spectrometry (GC-MS) to analyze the farnesol secretion in the supernatant when *C. albicans* cells were cultured with RAB. The data demonstrated that RAB stimulated farnesol production in concentration and time dependent manners (Fig. 6 A and B). Further investigation suggested the increased farnesol production was consistent with the induced Dpp3 expression by RAB (Fig. 6 C). These data imply RAB stimulates the expression of Dpp3, synthesizing more farnesol, which represses the Cdc35 activity and then inhibits the morphogenesis switch.

### Adhesins Reduced by RAB

Adhesins Hwp1, Als3 and Ece1 are associated with hyphae formation [32], [33]. Among the three proteins, Als3 is also an invasin, required for endocytosis by host cells [34]. qPCR results suggested that the transcript levels of *ALS3, HWP1* and *ECE1* were significantly reduced by RAB (Fig. 3 A). To determine the expression of Als3 protein in the presence of RAB, we constructed a strain with Als3-GFP, named MG1003. Due to the low expression of Als3, an immunofluorescence-like method was used to amplify the GFP fluorescent signal. Fluorescence intensity was measured using flow cytometry as well as fluorescence microscopy. We found that Als3 expression was markedly suppressed whether *C. albicans* cells were cultured in polystyrene substrate or KB/VCR cells (Fig. 7).

## Discussion

*Candida albicans*, a dimorphic fungal pathogen, undergoes yeast-to-hyphal transition. The hyphal form has the advantage of adhering and invading the tissue in the pathogenesis [35]. The formation of hyphae is also essential for specific iron acquisition from intracellular host sources and escaping from immune evasion [4], [36]. And dimorphism plays a critical role in pathogenicity at both superficial and systemic levels. Jacobsen et al. proposed that targeted inhibition of the yeast-to-hyphal transition or modulation of the immune response associated with dimorphism are very attractive options for controlling *C. albicans* infections in the future [37].

In *C. albicans*, the yeast-to-hyphal transition is positively regulated by Ras1-cAMP-Efg1 and MAPK signaling pathways [5]. cAMP is a strong regulator of *C. albicans* morphogenesis. Inhibition of cAMP synthesis blocks *C. albicans* in the yeast morphology under the majority of hypha-inducing conditions [38], suggesting that a certain level of cAMP is required for filamentation irrespective of the activated signaling mechanism. cAMP is synthesized by Cdc35 and *cdc35Δ/Δ* is avirulent in a mucosal membrane mouse model as well as a systemic infectious murine model [38]. Due to the critical importance of cAMP in hyphal development, Cdc35 and other regulators of the cAMP signaling pathway will be useful targets for antifungal drugs.

Lichen-derived RAB displays a moderate antifungal efficacy *in vitro* and *in vivo* (Table 2). More attractive for fighting fungal infection is that RAB could block the switch from yeast to hyphae, which results in the attenuated virulence of infected-nematodes (Fig. 2), consistent with the hypothesis that filaments contribute to the virulence [39]. To elucidate the molecular mechanism, we performed qPCR to examine the transcript levels of the pathways in regulating the morphogenesis switch. qPCR revealed the Ras1-cAMP-Efg1 pathway was downregulated by RAB and *PDE2*, encoding phosphodiesterase, was upregulated to hydrolyze cAMP. Because of the critical role of cAMP in signaling hyphae development, we measured the intracellular cAMP level following the treatment of RAB. The results showed the cAMP level was reduced at two different time points (8 h and 16 h) throughout the growth. At the same time, the addition of db-cAMP could restore the hyphae growth, suggesting the decreased cAMP level was the key point responsible for the retarded yeast-to-hyphal transition. Then we measured the factors affecting the cAMP synthesis. We focused on the Cdc35 that synthesizes cAMP. We found that the activity of Cdc35 in the cell lysates was inhibited when the cells were treated by RAB. However, RAB had no effect on the catalytic domain of Cdc35. These results indicated other factors induced by RAB might affect the Cdc35 activity. Previous studies have shown farnesol could directly inhibit the activity of Cdc35 and regulate the Ras1-cAMP-Efg1 pathway [30], [40]. Farnesol secreted by *C. albicans* cells was actually increased in the presence of RAB (Fig. 6 A and B). Based on the above analysis we propose that the stimulated production of farnesol by RAB represses the activity of Cdc35 which results in the defect of cAMP synthesis and thereby blocks the yeast-to-hyphal transition.

In sum, we elucidate the potential mechanism through which RAB inhibits the dimorphic switch of *C. albicans*. And our data provide an opportunity to expand the current antifungal agents to reduce the virulence of *C. albicans*.
